# Evaluation and management of pulseless pink/pale hand syndrome coexisting with supracondylar fractures of the humerus in children

**DOI:** 10.1007/s00590-013-1337-4

**Published:** 2013-10-15

**Authors:** Łukasz Matuszewski

**Affiliations:** Pediatric Orthopedic and Rehabilitation Department, Medical University of Lublin, Chodźki 2, 20-093 Lublin, Poland

**Keywords:** Supracondylar fractures, Vascular injuries, Pediatric

## Abstract

Elbow region fractures are the most common injuries in children. Among them, supracondylar fractures of the humerus are the most frequent. Massive displacement of the fractured bone causes severe injury to the soft tissue of that particular region. As a result, various types of injuries to the brachial artery such as entrapment, laceration, spasm of the vessel, and the presence of an intimal tear or thrombus formation are usually observed. The main goal of this study was to present our approach to children with supracondylar humerus fractures associated with brachial artery injuries. We would especially like to emphasize the necessity for other conservative or operative treatment concerning *pulseless hand* symptoms coexisting with supracondylar fractures of the humeral bone in children population. Data from 67 children were evaluated in our study. Supracondylar fractures were classified according to the Gartland’s scale. All patients had displaced extension type III injuries. During our follow-up study, we used Flynn’s grading system to evaluate functions of the elbow joint, forearm and wrist. Mean follow-up was 18 months; range, 13 months to 4 years. In the follow-up study, very good or good results were achieved in all 32 patients treated conservatively together with 6 patients with *pulseless pink hand* symptom. Very good or good results were achieved in 88 % of 35 patients operated on. Children who, after satisfactory closed reduction, have a well-perfused hand but absent radial pulse do not necessarily require routine exploration of the brachial artery. Conservative treatment should be applied unless additional signs of vascular compromise appear. Thus, exploration of the cubital fossa should be performed only if circulation is not restored by closed reduction.

## Introduction

Elbow region fractures are the most common injuries in children. Among them, supracondylar fractures of the humerus are the most frequent. Massive displacement of the fractured bone causes severe injury to the soft tissue of that particular region, i.e., arteries and nerves. Though diagnosis and treatment seem to be straightforward, effects of potential errors or oversights are irreversible. The most popular classification of supracondylar fractures of the humeral bone in children is that presented in 1959 by Gartland [1, 2]. Grade III supracondylar fractures (according to Gartland’s classification) are the results of a high-energy injury. The main mechanism of injury is a large displacement of the proximal fractured fragment in an “extension type” fracture and the nearness of soft tissue elements. As a result, various types of injuries to the brachial artery such as entrapment, laceration, spasm of the vessel, and the presence of an intimal tear or thrombus formation are usually observed [3]. In nerve injuries, the median nerve is most affected. Lesions of the radial nerve that are caused by posteromedial displacement are less frequent [2]. The majority of nerve damage manifests as neurapraxias and therefore resolves spontaneously [4].

Treatment for supracondylar humeral fractures in children used to be non-problematic. Nowadays, complications following supracondylar fractures are extremely rare. The most important complication is one described in the nineteenth century by Volkmann.

Exploration of the cubital fossa is required in fractures with permanent blood flow disturbance in the distal part of the upper limb (pulseless, pale and cold hand*).* In such cases, specific signs of vascular compromise and poor blood perfusion are observed as pain on a passive stretch of the wrist, absence of capillary refill, failure to record oxygen saturation and lowering of the hand’s temperature [5].

## Aim of the paper

The main goal of this study was to present our approach in children with supracondylar fractures of the humerus associated with vascular injuries. It is known that treatment of patients with a pink pulseless hand still remains controversial for some authors. Therefore, we would especially like to emphasize the necessity for other conservative or operative treatment concerning supracondylar fractures with coexisting brachial artery lesion.

## Materials and methods

**Fig. 1:**
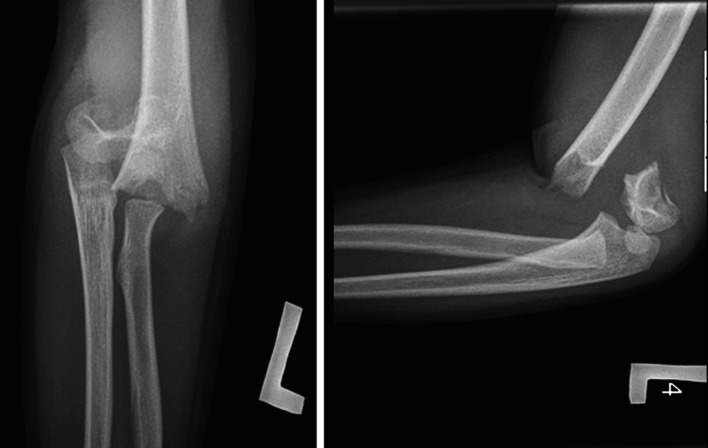
X-ray of third type of supracondylar humerus fracture

Research is based on the 1986–2011 data on patients treated in the Clinic of Pediatric Surgery and Traumatology and Pediatric Orthopedic and Rehabilitation Clinic Medical University of Lublin. During that period, 1,275 children were hospitalized with various elbow fractures. Six hundred ninety-five (54 %) of them were supracondylar fractures of the humerus. Data from 67 children [mean age 9.5 (range 4–17 years)] that included 46 male and 21 female patients were evaluated in our study. Supracondylar fractures were classified according to the Gartland’s scale. All patients had displaced extension type III injuries (Fig. 1). There were 7 compound fractures among them. During our follow-up study, we used Flynn’s grading system to evaluate functions of the elbow joint, forearm and wrist. Mean follow-up was 18 months and range 12 months to 4 years.

Perfusion disturbances were evaluated based on careful clinical examination in the emergency room. Capillary refill and oxygen saturation in the distal part of the injured limb were routinely checked to assess circulation to the extremity. Doppler ultrasound examination was used beginning in 2003 to assess a blood perfusion and morphology of the lesion. During statistic analysis, we used independent score statistics test. All statistical calculations were performed using 10.0 STATISTICA software (StatSoft, Poland).

## Results

**Fig. 2:**
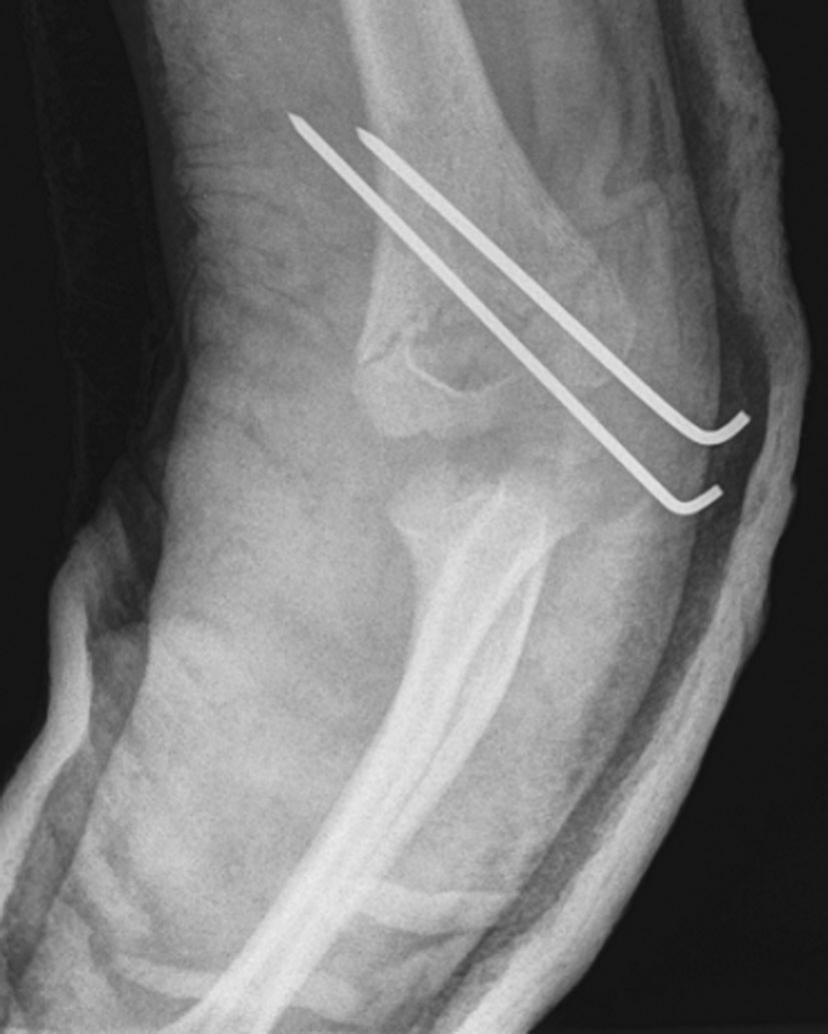
X-ray of third type of supracondylar humerus fracture after reduction—anteroposterior view

**Fig. 3:**
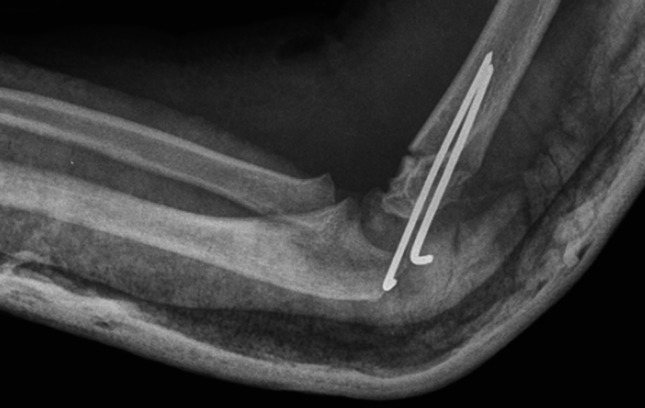
X-ray of third type of supracondylar humerus fracture after reduction—lateral view

The study included 67 patients with injuries of the arterials in supracondylar fractures of the humerus. The injuries were classified into defined categories. In 32 (47 %) patients, we observed a lack of radial pulse with cold and pale hand syndrome before reduction. In 26 of them, all those symptoms disappeared immediately after closed reduction. Radial pulse returned in a mean time of 25 min (range 2–65 min). In other 6, we noticed *pulseless pink hand* with lack of radial pulse after reduction. However, in all patients, proper capillary refill with sufficient oxygen saturation on the index finger of affected limb recovered immediately after anatomical reduction in the fracture. All those children were treated conservatively with good results no more than 3 h after the injury (mean 1 h and 10 min). Reduction and stabilization of the fracture by two K-wires (lateral or medial and lateral) were performed under general anesthesia and then confirmed by anteroposterior and lateral X-ray views (Figs. 2, 3). In the follow-up study, very good or good results (according to Flynn’s criteria) were achieved in all 32 patients. In those 6 patients with *pulseless pink hand* symptoms, radial pulse returned no later than on third day after injury. Good or very good vascular status was achieved confirmed by ultrasound examination of the artery during follow-up. No growth disturbances of the limb or some late vascular insufficiency was observed.

**Fig. 4:**
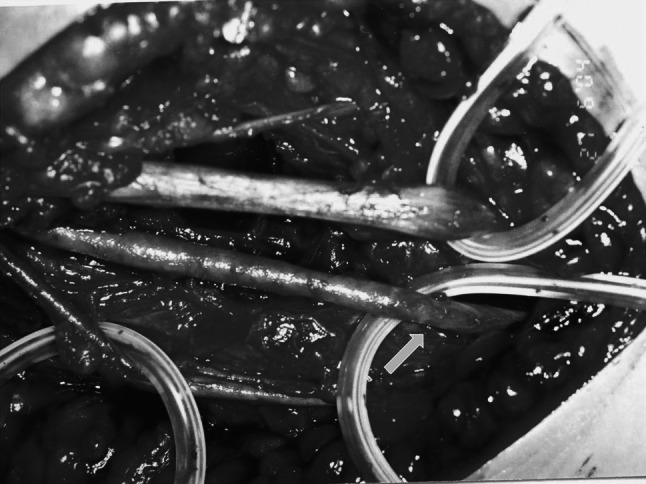
Adventitial hematoma formation together with brachial arterial spasm

**Fig. 5:**
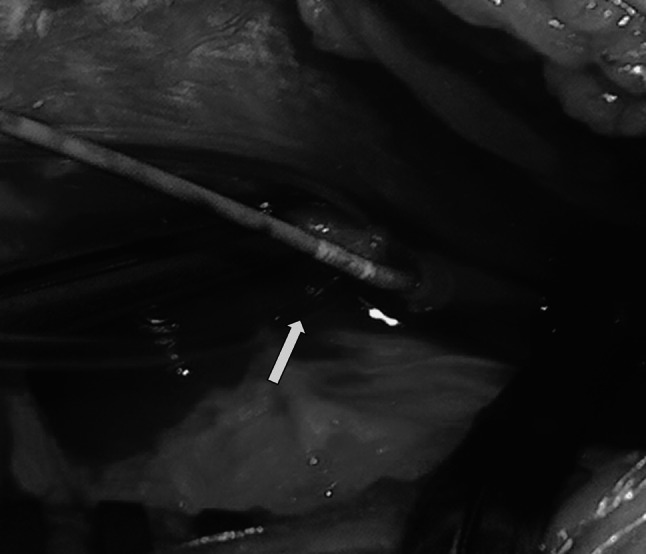
Using of Fogarty’s catheter in brachial artery thrombus evacuation

**Fig. 6:**
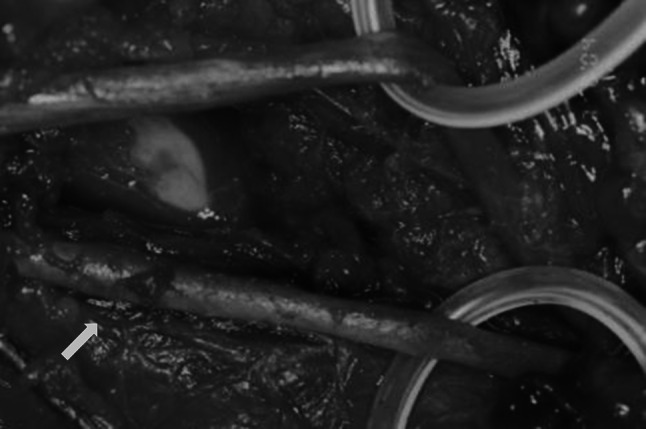
Isolated brachial artery contusion

The remaining 35 (53 %) patients were treated surgically (Table 1). Surgical exploration of the brachial artery was performed in 34 patients within no more than 3 h after injury (mean 2 h and 10 min). Pulseless, pale and cold hand syndrome with severe pain in the upper limb region was identified. In all of them, the initial attempt at a closed reduction was unsuccessful in restoring the radial pulse and proper capillary refill with oxygen saturation on the index finger of the affected limb. One patient was treated surgically because of vascular disturbances. That procedure took place 8 h after the injury due to a delayed transfer of the patient from another clinic. Exploration of the cubital fossa is always performed by the standard anterior approach through an “lazy S” incision. During surgery of the brachial artery of 16 patients, associated nerve contusions were noticed, 12 median and 4 radial. In 10 of those patients, adventitial hematoma formation together with arteriospasm was found (Fig. 4). The procedure began by performing osteosynthesis of the humerus followed by the removal of the hematoma and the adventitia in a long part of the artery together with nerve decompression. In all patients, the spasm was released, a radial pulse was quickly restored, and nerve function recovered. In 6 patients, brachial artery occlusion (because of thrombus formation) was observed. Evacuation of the arterial thrombus was performed. Then, arterial lumens were checked using Fogarty’s catheter and flushed with a low molecular weight anticoagulant drug (Fig. 5). This procedure led to full restoration of a palpable radial pulse in a short time. In the other 6 patients, with poor perfusion status during surgery, an isolated brachial artery contusion was found, with the median nerve intact (Fig. 6). To restore proper circulation, the adventitia was removed.

**Table 1:** Type of operations and morphology of brachial artery injury in patients with supracondylar fractures of the humerus bone

Type of operation	Morphology of the injury	Number of patients
Osteosynthesis of the humerus bone, removal of the hematoma and the adventitia. Nerve’s decompression	Adventitial hematoma formation and arterial spasm, nerve contusion	10
Osteosynthesis of the humerus bone, evacuation of the artery thrombus. Using of Fogarty’s catheter with low molecular weight anticoagulant drug flushing. Nerve’s decompression	Artery occlusion (because of thrombus formation), nerve contusion	6
Osteosynthesis of the humerus bone, removal of the adventitia	Isolated brachial artery contusion	6
Osteosynthesis of the humerus bone, anastomosis with a synthetic, non-absorbable monofilament suture (6–0 polypropylene)	Partial artery rupture	6
Osteosynthesis of the humerus bone, resection and anastomosis of the brachial artery with/without vein graft	Complete rupture of the artery	4
Osteosynthesis of the humerus bone, using of Fogarty’s catheter to release the spasm	Arterial spasm in a segment of the artery	3
All		35

In 6 other patients, a partial arterial rupture was noticed. Anastomosis with a synthetic, non-absorbable monofilament suture on an atraumatic needle (6–0 polypropylene) was applied. In the other 4 children, complete rupture of the artery was found and it was repaired using standard procedures. These include debridement of the lacerated vessel and assuring a lack of tension in the anastomosis, which contribute to the success of this procedure. In 2 cases, anastomosis with a vein graft was necessary because of extensive brachial artery injury. In the last 3 patients, vascular disturbances were caused by arteriospasm in a segment of the artery. After exploration of the brachial artery, Fogarty’s catheter was used to help release the spasm and restore proper vascular status.

According to Flynn’s grading system, very good or good results were achieved in 91.5 % of patients operated on. We analyzed bone healing, elbow function and neurovascular integrity. The reduction in the fracture was considered to be in anatomical position in almost all of operated children (93.8 %) and satisfactory in 2 (6.2 %) patients. All fractures healed within a mean of 4.1 weeks (range 3.6–5.1 weeks). Full limb movement together with a very good or good vascular status was achieved. No decreased growth of the limb or vascular insufficiency with exercise was observed.

In 3 (8.5 %) patients, results were poor or bad. One child had symptoms of myositis ossificans with severe limitation of movements in the elbow joint. The other, who was admitted to the clinic 8 h after injury, had Volkmann’s contracture. The third child, who suffered multi-organ trauma together with complete rupture of the brachial artery, had insufficient blood circulation in the distal part of upper limb, which caused muscle atrophy with decreased growth and poor limb function. A vein graft used during a 4-year follow-up did not improve his conditions.

## Discussion

Supracondylar fracture of the humeral bone is one of the most common injuries of the children’s organs of movement. The frequency of the supracondylar fractures of the proximal part of the humeral bone, as compared to other injuries of an elbow joint, was evaluated by Lauer and Gehling at a level of approximately 60 % [6, 7]. Vascular insufficiency effected 3–11 % of patients [8]. This statistic is similar to our data. Recently the number of surgical intervention in supracondylar fractures of the humerus with arterial or nerves injuries has decreased [9, 10]. Such a downward trend was also observed in this study that involved operations on 20 children within first 11 years (1986–1996) and only on 12 children within the following period of 15 years (1997–2011). The main causes of such progress are the development of better diagnostic tools and an improvement in methods of treatment (power Doppler ultrasound or C-arm X-ray).

A supracondylar fracture requires an orthopedic consultation for the determination of appropriate intervention. Most pediatric orthopedists recommend closed reduction and percutaneous pin fixation [11–14]. Because supracondylar fractures of the humerus in children should be considered a surgical emergency, the immediacy of admission to a clinic is of the prime importance. This is a crucial factor, especially in case of the third type of fracture with any ischemic disturbances of the distal part of the upper limb. In this study, most of the patients demonstrating considerable ischemic disturbances were treated no more than 6 h after the injury. In such cases, the vascular discrepancy was identified on the basis of clinical examination, capillary refill and oxygen saturation test, and using Doppler ultrasound. The anatomical reduction in the fracture is very important. The reduction in the fracture was considered to be in the anatomical position in all non-operated children, and it is believed that it was crucial for restoring proper blood perfusion. This procedure complies with that of the others [15–17].

Lack of radial pulses and a cold, pale hand after closed reduction and percutaneous pinning of supracondylar fractures necessitated exploration of the cubital fossa [18, 19]. Korompilias et al. [3] noticed that a delay in determining the nature and the extension of the vascular injury with time-consuming imaging studies was probably unnecessary. According to Sabharwal et al. [8], conventional angiography or MRA, both technically feasible and safe, had a high rate of asymptomatic reocclusion and residual stenoses of the brachial artery. However, Luria et al. [17] concluded that, if the radial pulse did not return after reduction in the fracture, an exploration of the cubital fossa was recommended only if intra-operative angiographic evaluation revealed a brachial artery injury. They noted that angiography is a helpful procedure that may prevent unnecessary exploration of the brachial artery, as in the case of arterial spasm. In the presented studies, an angiography was not used similar to the report shown by Shaw et al. [20]. However, according to Korompilias et al. [3], angiography may not be sufficient for distinguishing arterial spasm from an intimal tear. In their material, persistent arterial spasm required removal of the adventitia.

In this study, morphology of the injured brachial artery was variable. Vascular lesions varied from simple contusion with arteriospasm to severe ruptures of a vessel. Now, advances in vascular surgery allow treating rare and severe cases. All methods of treatment used in this study lead to good or very good results. Such findings agree with observations of other authors [1, 6, 21].

In third type of supracondylar humeral fracture, lesions of the vessels and nerves, as well as combinations of both, must be always taken into consideration. Luria et al. [17] found a statistically significant correlation between the median nerve injury and the brachial artery lesion. However, they did not notice a correlation between the type of a vascular lesion and nerve injury. In the presented material, more than a half of patients with vascular injury (53 %) had nerve injury. All injuries seemed to occur due to tenting or entrapment of the nerve on the sharp dislocated proximal humeral fragment. Campbell et al. [2] conclude that posterolateral displacement is strongly associated with median nerve injury; posteromedial displacement is responsible for radial nerve injury. In this study, only median and radial nerves were affected. In almost all operating cases, contusion of a nerve (causing its neuropraxy) was found. A regression of those injuries was achieved under conservative treatment that is also recommended by others reports [4, 22]. Total ruptures of the nerve are very rare; however, in the presented material, one patient suffered such injury.

Luria et al. [17] analyzed vascular complications of 24 children with supracondylar fractures of the humerus. In their research, reduction in the fracture was followed by a return of the pulse in 58 % of cases, similarly to this study (53 %). Considering the presented data, urgent exploration of the cubital fossa is always necessary in a child with a pulseless, pale and cold hand, if these symptoms were not relieved by reduction in a supracondylar fracture of the distal humerus. Although complications sometimes occur, a long-term prognosis for a successful outcome is very good, if the fracture is appropriately treated on time [7, 16]. Results show that exploration of the cubital fossa is a safe and effective method of managing these injuries.

Nowadays complications are very rare and mainly caused by a delay time of surgical intervention [11, 23, 24]. However, opinions vary on a detailed diagnosis and treatment of vascular compromise in cases where the hand remains pulseless but well perfused [3, 8, 15]. Some authors are of the opinion that an absence of pulse is an indicator of arterial injury, even if the hand appears well perfused. They suggest vascular exploration and repair in selected cases [3, 25, 26]. Observation is the treatment of choice for many authors [18, 21, 23, 27], including that of this paper. Absence of the radial pulse immediately after reduction but with coexisting good hand perfusion led us to very good outcomes. No limb length discrepancy, claudication, cold intolerance or thrombus migration during follow-up were noticed.

The results of the study allow for the drawing of the following main conclusions:

Children who, after satisfactory closed reduction, have a well-perfused hand but absent radial pulse do not necessarily require routine exploration of the brachial artery. However, anatomical reduction in the fracture is mandatory. Conservative treatment should be applied unless additional signs of vascular compromise appear. Thus, in case of blood circulation disturbance, the exploration of cubital fossa should be performed only if circulation is not restored after closed reduction. In such case, surgical exploration of the artery is recommended.
